# RScan: fast searching structural similarities for structured RNAs in large databases

**DOI:** 10.1186/1471-2164-8-257

**Published:** 2007-07-31

**Authors:** Chenghai Xue, Guo-Ping Liu

**Affiliations:** 1 MOE Key Laboratory of Bioinformatics and Bioinformatics Div, TNLIST/Department of Automation, Tsinghua University, Beijing 100084, China; 2 Department of Engineering, University of Glamorgan, Pontypridd CF37 1DL, UK; 3 LCSIS, Institute of Automation, CAS, Beijing 100080, China

## Abstract

**Background:**

Many RNAs have evolutionarily conserved secondary structures instead of primary sequences. Recently, there are an increasing number of methods being developed with focus on the structural alignments for finding conserved secondary structures as well as common structural motifs in pair-wise or multiple sequences. A challenging task is to search similar structures quickly for structured RNA sequences in large genomic databases since existing methods are too slow to be used in large databases.

**Results:**

An implementation of a fast structural alignment algorithm, RScan, is proposed to fulfill the task. RScan is developed by levering the advantages of both hashing algorithms and local alignment algorithms. In our experiment, on the average, the times for searching a tRNA and an rRNA in the randomized *A. pernix *genome are only 256 seconds and 832 seconds respectively by using RScan, but need 3,178 seconds and 8,951 seconds respectively by using an existing method RSEARCH. Remarkably, RScan can handle large database queries, taking less than 4 minutes for searching similar structures for a microRNA precursor in human chromosome 21.

**Conclusion:**

These results indicate that RScan is a preferable choice for real-life application of searching structural similarities for structured RNAs in large databases. RScan software is freely available at .

## Background

A wide range of RNA molecules can form specific secondary structures by folding their primary sequences. RNA secondary structures play important roles in cellular processes, such as regulating gene expressions and producing non-coding transcriptional products [1,2]. The secondary structures of many non-coding RNAs (ncRNA), like the cloverleaf structure of tRNA and the hairpin structure of microRNA precursor (pre-miRNA), have been evolutionarily conserved instead of the primary sequences [3].

In recent years, several computational methods have been reported to find these conserved secondary structures, as well as common local structural motifs in pair-wise or multiple sequences. The programs QRNA [4], ddbRNA [5] and MSARI [6] were developed, one after another, to detect functional ncRNAs with conserved structures. Washietl *et al*. implemented program RNAz [7], which led to a mapping of thousands of conserved structural and functional RNA in the human genome [8]. In addition, the local structures or structural motifs of RNA molecules were even more important in RNA function study. Macke *et al*. defined specific types of RNA motif and developed RNAMotif to search the structural elements [9]. The program ERPIN was based on the secondary structure profile and used the RNA sequence alignment with secondary structure information for motif definition and identification [10]. Hoechsmann *et al*. utilized tree alignment and forest alignment to implement local similarity comparison in RNA secondary structures [11]. The Vienna RNA package was probably a comprehensive RNA secondary structure prediction and comparison tool [12], and RNALfold was designed to predict locally stable RNA structures in single genome [13]. Havgaard and co-workers focused on detecting the common local structures between two RNA sequences with low sequence similarity [14]. A similar procedure RNAProfile was used for detecting conserved structural motifs in unaligned RNA sequences [15]. Recently, Liu *et al*. reported RSmatch for aligning RNA secondary structures and motif detection [16], which used a tree model to organize the structure components. For a comprehensive comparison, Freyhult *et al*. assessed the effectiveness of 12 methods that can perform RNA homology search. The result showed that most of them have low accuracy [17].

We now face a challenging task: given a RNA sequence with secondary structure, how to find structural homologs in a large genome database effectively. To deal with this task, Klein and Eddy developed a pair-wise alignment algorithm RSEARCH [1] based on the profile stochastic context-free grammar. RSEARCH used the base pair and single nucleotide substitution matrix RIBOSUM to find optimal structural alignments between a RNA sequence and a sequence database. It succeeded in searching in *Archaeals*, yeast and *Arabidopsis thaliana *databases. However, the time complexity of the algorithm is *O*(*NM*^3^), where *N *is the length of the database sequence and *M *is the length of the query sequence. RSEARCH is very slow on a personal computer (PC) [1,18]. Subsequently, Weinberg and Ruzzo used a rigorous filter to eliminate the sequences that provably could not be annotated as homologs of known ncRNA gene family in the genome database [19,20]. Then, they achieved a fast search when annotating the new members of known ncRNAs in the genome. Using a similar strategy, Bafna and Zhang invented FastR [18], which was faster than RSEARCH by dividing the search into two steps. Firstly, FastR filtered a large proportion of the database according to the analysis of the structural element and sequence information of the query RNA, and then, the searching was run on the remaining of the database. The query time of FastR drastically decreased due to a smaller search database produced by the filtering process. However, since FastR must filter the database for each specific query, it was actually an invalid comparison without considering the large overhead required to perform the filtering step. Moreover, FastR also lost sensitivity due to the filtering [18].

In this paper we focus our attention on finding structural similarities for a structured query RNA in a large database efficiently and quickly and propose an algorithm RScan to do the job. These conserved structural similarities are possible functional homologs of the query RNA. RScan is developed upon the basis of SSAHA [21] and the Smith-Waterman [22] algorithms. RScan carries out the search in two steps. Firstly, it builds a hash table for a database. It then searches structural similarities with the hash table for a query in the database. Since the hash table is generated only once for searching any query in a given database, this can drastically reduce the time required to perform a search. Six ncRNA datasets were used as a test-case to make a comparison between RScan and RSEARCH, and the results were encouraging. Moreover, RScan and RSEARCH obtained a close identification rate when searching for a tRNA and an rRNA query in a randomized genome. RScan took only 256 seconds (s) versus 3,178 s with RSEARCH for tRNA. rRNA yielded 832 s with RScan versus 8,951 s with RSEARCH. The experiment of searching structural similarities for a pre-miRNA in human chromosome 21 took less than 4 minutes. RScan makes a good searching tool when queried database is large.

## Results

### Pre-processing and definitions

**Figure 1:**
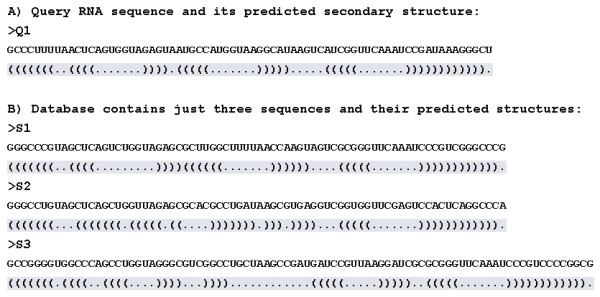
**Example of the structural query and the structural database for RScan search**. A) The shadowed symbol sequence is defined as the "structural query". B) The set of shadowed symbol sequences is defined as the "structural database".

We illustrate the preprocessing of a query and a database with a simple example. The query sequence is shown in part A of Figure 1. The secondary structure of the query sequence is predicted using RNAfold [23]. In the predicted secondary structure, there are only two statuses for each nucleotide, paired or unpaired, indicated by the symbols "(" or ")" (paired case), and "." (unpaired case). The symbol sequence composing of "(", ")" and "." is defined as the "structural query", shown in line 3 of Figure 1A. The example database contains only three sequences, which are shown in part B of Figure 1. The secondary structures of the sequences are also predicted using RNAfold [23]. The set of all symbol sequences of secondary structures is defined as the "structural database", which are shadow parts of Figure 1B.

### Search algorithm of RScan

RScan directly searches optimal structural alignments between a structural query and a structural database. RScan is based on SSAHA algorithm [21], which utilizes the hashing algorithm to perform a fast search for large genome databases, and the Smith-Waterman algorithm [22], which is a basic local alignment algorithm.

#### Construct the hash table for a structural database

**Figure 2:**
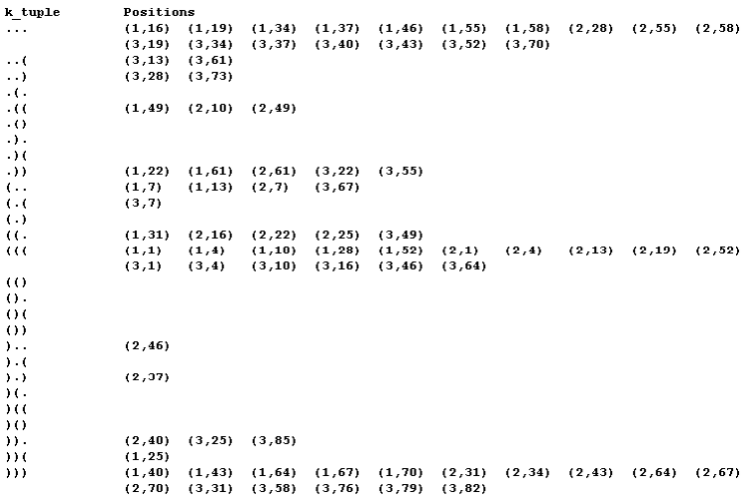
**A *3*-tuple hash table for the structural database**. The *k*-tuple window moves by the size of the *k*-tuple when handling the structural database. Each occurrence position of *k*-tuple in the example structural database is represented by a two-dimension vector, the first is the symbol sequence number and the second is the position in corresponding symbol sequence.

RScan breaks the symbol sequences in a structural database into consecutive *k*-tuples of *k *contiguous symbols and stores the positions of each occurrence of *k*-tuple using the hash table. Figure 2 shows the hash table of the example structural database in the case of *k *= 3.

#### Search the structural query

**Figure 3:**
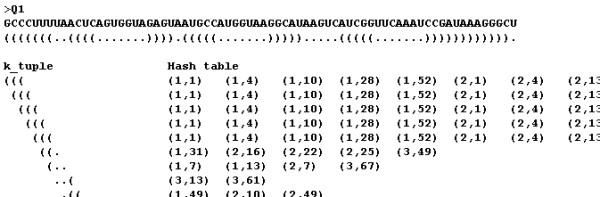
**Illustration for part of *3*-tuple of the structural query retrieving the hash table entries**. The *k*-tuple slide window moves 1 nt per step when handling the structural query. Note that the figure is abridged.

Each *k*-tuple occurring in the structural query has corresponding entries in the hash table. The example structural query in the case of *k *= 3 is shown in Figure 3. Using the same strategy with SSAHA, RScan sorts these entries to obtain contiguous matching symbols over a given threshold, which is called the "match-core". The Smith-Waterman algorithm is then used to obtain an optimal structural alignment between the structural query and the structural database along two sides of the match-core.

#### Scoring function

RScan uses a binary match\unmatch score function to calculate the score of each alignment. The penalty values of the match, unmatch and the insertion\deletion gap (indel) can be adjusted on demand. In this paper, penalty values are set as: match = 1, unmatch = -2, indel = -1.

#### Alignment output

**Figure 4:**
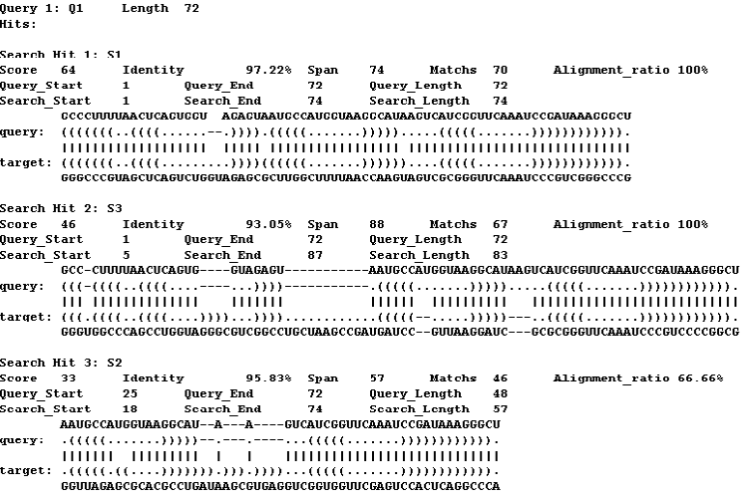
Output format of RScan.

RScan searches for the structural query within the structural database and reports significant structural alignments according to the user's requirements. Figure 4 shows the results for the example. It should be emphasized that the query does not have sequence similarity with the sequences in the database, which is calculated using BLASTCLUST [24] with parameters S = 80, L = 0.5, W = 16.

The output reports three structural alignment hits. The first hit is an accurate alignment between the structural query and the structural S1, which just contains two mismatches and two indels in whole alignment. Hit 2 is also a good structural alignment though more indels occur and the lengths of the query (72 nucleotides) and the hit S3 (87 nucleotides) are different, shown in Figure 1. In hit 3, RScan only finds a local structural alignment between the query and S2. Two parameters, "Alignment_ratio" and "Identity", should be introduced more carefully. Using hit 1 as an example, the number of the aligned symbols of the query is 72 and the length of the query is 72, so the parameter "Alignment_ratio" is 100% (72/72). And there are 70 matches in the aligned symbols of the query, so "Identity" is 97.22% (70/72). Users can set the thresholds for "Alignment_ratio" and "Identity", and RScan only reports the alignments which are greater than the thresholds. In following experiments, we set thresholds for finding significant global alignments.

**Figure 5:**
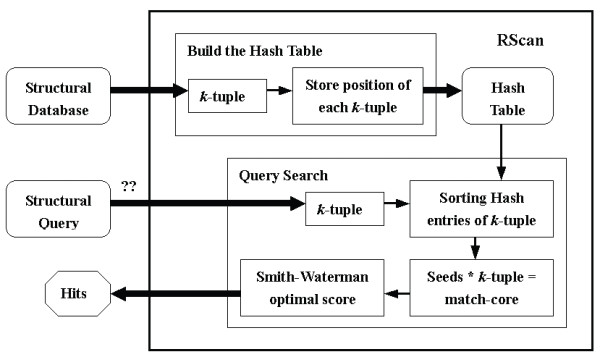
RScan flow.

The RScan procedure is shown in Figure 5. The difference between SSAHA and RScan is that SSAHA processes the genomic sequence itself, whereas RScan processes the symbol sequence.

### Comparisons between RScan and three other methods: Blast, RSEARCH and RSmatch

Several sets of data were used to evaluate the performance of RScan. The datasets were six ncRNA families, one tRNA, two rRNA and three RNase P families, from Rfam [25], whose member sequences were filtered using proper steps, for example, eliminating the sequences with high sequence similarity (see details in Methods). For each filtered dataset, one member was used as the query and the other members were treated to build the database. RScan and RSEARCH performed the searches on these datasets and compared the accuracy and the speed.

First of all, it should be emphasized that Blast can not find sequence homologies between the query and the sequences in the datasets because of the filtering step (see Methods). By comparison, RScan and RSEARCH recognized numerous significant structural similarities for the query. This indicates RScan is more sensitive than Blast for structural alignment.

For each dataset, in Table 1, RScan found the most of true structural similarities for a structural query. On RF00011, the identification rate was 100% and on other families, the identification rates were from 58% to 83%. RSEARCH achieved better identification rates than RScan on these datasets. It recognized all true similarities on four families and achieved the identify rates of 85% on RF00010 and 82% on RF00177, respectively. However, RScan could detect more alignments when tuning the parameters. RScan found 49 tRNA hits and the identification rate is 74% for the query under "Identity ≥ 90% and Alignment_ratio ≥ 95%". When the parameters were set as "Identity ≥ 85% and Alignment_ratio ≥ 90%", RScan found 56 true tRNA similarities, and the identification rate rose to 85%. Moreover, on RF00001 dataset, RScan recognized 20 hits when the parameter *k *set as "*k *= 7". If "*k *= 5", RScan detected all twenty-four true similarities. Properly tuning thresholds for RScan parameters was depending on the demands. Our experiments sought for highly significant structural similarity for the query, so the thresholds were strict. All detailed parameters of each test were provided in the supplementary materials.

**Table 1:** Comparison of RScan and RSEARCH on the ncRNA family datasets

**Family**	**Query**		**Database**		**Methods**	**Hits/TP/FN^2^**	**Time (h/m/s^3^)**
				
	**Rfam ID**	**Length (nt)**	**Size (nt)^1^**	**# of True**			
tRNA RF00005	AB042240.3/15036-15107	72	4,851	66	RSEARCH	66/66/0	6 m 3 s
					RScan	49/49/17	5 s
rRNA RF00001	M28193.1/1-119	119	2,852	24	RSEARCH	24/24/0	26 m 28 s
					RScan	20/20/4	6 s
ribozyme RF00030	AY090594.1/1-274	274	2,422	8	RSEARCH	8/8/0	6 h 47 m 47 s
					RScan	6/6/2	10 s
ribozyme RF00011	U64878.1/1-308	308	2,626	7	RSEARCH	7/7/0	10 h 24 m 32 s
					RScan	7/7/0	27 s
ribozyme RF00010	U28101.1/1-327	327	18,642	52	RSEARCH	48/44/8	12 h 17 m 58 s
					RScan	30/30/22	3 m 9 s
rRNA RF00177	AF050603.1/1-475	475	17,382	34	RSEARCH	31/28/6	41 h 3 m 27 s
					RScan	22/22/12	4 m 55 s

^**1 **^The size of database is the total nucleotides of each database. nt: nucleotides.

^**2 **^TP: true positive; FN: false negative.

^**3 **^h/m/s: hour/minute/second.

The last column of Table 1 listed the computing times of RScan and RSEARCH on six datasets. RScan was remarkably faster than RSEARCH. RScan only took 5 seconds (s) and 6 s for searching a structural query in the structural RF00005 and RF00001 databases, respectively. In contrast, RSEARCH ran 363 s and 1,588 s for the same structural searches. On the three ribozyme families, RSEARCH needed 6~12 hours to finish the searches, but RScan took only 10 seconds to 3 minutes to do so. For searching RF00177 dataset, the most time consuming case, RSEARCH ran more than 41 hours, whereas RScan ran less than 5 minutes. In this case, the identification rate 82% of RSEARCH was 1.26 times higher than that of RScan, but the computing time of RSEARCH was 501 times than that of RScan.

The time complexity of RSEARCH is *O*(*NM*^3^), where *N *is the length of the database sequence and *M *is the length of the query sequence [1,26]. It will be very slow when *M *or *N *is large. Comparatively, RScan transfers the complicated similarity search problem of RNA secondary structure into a sequence alignment problem between a structural query and a structural database. So, the time complexity of RScan is *O*(*NM*), which is identical to the sequence alignment algorithms.

We also tested another RNA-specific search method, RSmatch [16]. It only found 19 accurate tRNA alignments on tRNA dataset and missed all hits on other five datasets (see supplementary materials).

Finally, in the experiments RScan showed higher sensitivity than Blast and RSmatch. Although RScan may loss some sensitivity compared with RSEARCH, it is greatly faster than RSEARCH. Actually, some real-life tasks do not need to retrieve all hits for a query. For example, an unknown query is asked if it belongs to a known ncRNA families. We can run a structural similarity search on the entire Rfam database. RScan will give a quick answer with sufficient sensitivity.

However, it should be noted that only those examples, whose predicted structures were satisfied the filtering criteria (see Methods), were used in the tests shown in Table 1. For example, when the dataset was constructed, part of tRNAs without typical cloverleaf structure were filtered. RScan was unable to accurately search a tRNA with two stem-loops in the database composed of tRNAs with three stem-loops. We therefore needed to study the impact of the accuracy of structure prediction.

For instants, there were totally 1114 records of tRNA family in Rfam seed data. According to the loop number (1~4) of each tRNA's predicted secondary structure, these tRNAs were classified as four groups. For each group, we used one tRNA as the query and other tRNAs to build database. RScan performed structural aligning on each group. There were 356 (32%) tRNAs that were predicted as the cloverleaf structures. RScan achieved 66% accuracy on the group with 3 loops. But the corresponding accuracy on entire tRNA family was only 21.1% (Table 2). This showed that RScan was greatly limited by the accuracy of structure prediction. We will have more discussion about it later. For a real-life searching task, there are two determinants: one is the accuracy of structure prediction and the other is the performance of RScan. In this paper, we are only able to deal with the latter.

**Table 2:** Impact of the accuracy of structure prediction

**Loops**	**tRNA Query**	**Database (Loops)**	**Database (Total)**	**Hits**	**% TP (Total)**
1	AF347001.1/16015-15948	160	1110 (excluding 4 queries)	25	2.3
2	D12694.1/2745-2677/	583		93	8.4
3	AB042240.3/15036-15107	355		234	21.1
4	AC024995.8/165717-165798	12		9	0.8

### Comparison between RScan and RSEARCH on a randomized genome

A randomized genome was created through shuffling a whole *A. pernix *genome while preserving di-nucleotide frequency. tRNAs or rRNAs were then inserted in the randomized genome. RScan and RSEARCH searched the tRNA or rRNA query in the randomized genome to evaluate the false positive and the false negative.

When searching tRNAs, RSEARCH found 57 hits, including 43 true tRNAs and 14 false hits, shown in Table 3. RScan detected 46 hits; among them 45 were true positive. When searching rRNAs, 19 of 31 hits detected by RSEARCH were true rRNAs, whereas 18 of 32 hits recognized by RScan were true. In the aspect of the computing time, RSEARCH ran 3,178 s and 8,951 s for searching the tRNA and the rRNA in the randomized genome on a PC. In comparison, RScan only took 256 s and 832 s for searching the structural tRNA in the 75 nt structural database and the structural rRNA in the 120 nt structural database, respectively. These illustrate that RScan achieves similar sensitivity as RSEARCH does, but only takes a fraction, less than ten percent, of the computation time of RSEARCH for the randomized genome search.

**Table 3:** Comparison of RScan and RSEARCH on the randomized genome

**Family^1^**	**# of True**	**Methods**	**Hits/TP/FN/FP^2^**	**Time**	**T_hash_^3^**	**T_search_^3^**
tRNA RF00005	49	RSEARCH	57/43/6/14	52 m 58 s	-	-
		RScan	46/45/4/1	4 m 16 s	7 s	4 m 9 s
rRNA RF00001	20	RSEARCH	31/19/1/12	2 h 29 m 11 s	-	-
		RScan	32/18/2/14	13 m 52 s	8 s	13 m 44 s

^**1 **^The tRNA sequences and rRNA sequences are inserted in the randomized genomes.

^**2 **^TP: true positive; FN: false negative; FP: false positive.

^**3 **^**T**_**hash**_: the time required by building the hash table; **T**_**search**_: the time required by searching.

The time used by RScan is composed of two parts: the time spent in building the hash table (T_hash_) and in searching (T_search_), like SSAHA [21]. T_hash _is inconsequential since the hash table is generated only once for a given database. The last two columns of Table 3 list T_hash _and T_search _in this experiment. It should be noted that RSEARCH searches the sequence database directly, but RScan searches a processed structural database, and this pre-processing step takes additional time. The times for generating the 75 nt and 120 nt structural database were 534 s and 510 s, respectively. Fortunately, the pre-processing to generate the structural database takes only once.

There another algorithm FastR, which is similar to RScan, comprises of two steps [26]. FastR performs the search on a filtered database, which is only a small proportion of a given genome. However, the search time is genome specific and depends on the efficiency of the filtering step in each query. Since FastR must filter the genome every time for each specific query, the search time should include the time spent on the filter step, which is in the range of hundreds of seconds for filtering the *A. pernix *genome [18]. By comparison, the computation time of RScan consists of T_hash _and T_search_. T_hash _is related to the database and is taken only once. Once the hash table is generated, it can be used for searching any query. T_search _is linear to the size of the database and the length of the query. So, RScan is a more efficient search strategy.

On the other hand, the experiments in Table 3 also reveal the limitation of RScan's application on the genome. A window with pre-chosen width probably leads to great changes of tRNAs' or rRNAs' predicted secondary structures. This is the main reason why RScan missed several tRNAs or rRNAs. When applying to native *A. pernix *genome, RScan displayed a bad performance for finding tRNAs or rRNAs. The reason is that it is difficult to give a proper window width for a genome. At the same time, it is impractical to build a huge structural database using windows with different widths. A promising solution is using RNALfold [13] to find stable structures or significant local structural motifs in a genome, and then using RScan to align the queries.

### Performance analysis of RScan with different parameters

In RScan parameter *k *creates *k *contiguous symbols ("(", ")" and ".") and it affects the search speed. Table 4 shows the results of searching rRNA query in the randomized genome with different *k*. It can be seen that the T_search _is approximately an inverse function of the parameter *k*.

**Table 4:** Performances of RScan with different *k *and match-core

***k***	**match-core**	**# of True**	**Hits/TP/FN/FP**	**T_hash _(s)**	**T_search _(s)**	**Time (s)**
7	14	20	32/18/2/14	8	824	832
9	18		15/11/9/4	4	60	64
11	22		11/10/10/1	17	6	23
		
7	21^1^		11/10/10/1	8	18	26

^**1 **^This match-core is 3 times of *k *here and other match-cores are 2 times of corresponding *k*.

Although a greater *k *reduces the search time, a large value *k *should not be selected solely upon this criterion. The combination of parameter *k *and parameter "match-core" determines the alignment, which directly affects the hit sensitivity. For example, in Table 4, when *k *is 7 and "match-core" is 2 times *k*, a candidate alignment is required to have at least 14 contiguous matching symbols; and then, this candidate is scored using the local alignment algorithm to obtain the final alignment. With *k *or match-core increasing, the candidates that satisfy the threshold of the match-core are decreasing. RScan found few alignments when the match-core is 22. So, the parameters should be properly selected to balance between the speed and the alignment sensitivity.

In addition, the hash table can be generated in advance, and used for searching any query. This is important and efficient when the database is large. Parameter *k *determines the storage size of the hash table. The storage contains two parts: hash keys and values. Values are the positions of occurrences of *k*-tuples in the structural database and the keys point to these values. With *k *increases, the storage requirement of the hash table decreases. In Table 5, for the 120 nt structural database, when *k *was 7, 9 or 11, the size of the hash table was 1.85 Mb, 1.44 Mb or 1.14 Mb, respectively.

**Table 5:** Time and Storage requirements for building hash table in advance

***k***	**match-core**	**T_hash_^1 ^(s)**	**T_search_^2 ^(s)**	**Hash Table Storage (Mb)**
7	14	10	820	1.85
9	18	6	57	1.44
11	22	19	5	1.14

^**1**^**T**_**hash **_contains the times required by building the hash table and writing the hash table into the hard disk.

^2^**T**_**search **_contains the times required by reading the hash table generated in advance into the computer memory and searching.

It should be noted that the computation time in Table 5, the case that the hash table was generated in advance, and the computation time in Table 4, the case that the hash table was not generated in advance, are slightly different. T_search _in Table 5 was slightly less than T_search _in Table 4. The reason is that the number of keys in the hash table was reduced; there is a compression step in the former case that rids of the keys that refers to null values. Searches are carried out on all keys loaded in memory for the latter case, which implies a time waste on key comparisons during hash lookup for invalid entries. And the additional time spent on reading the hash table pre-built into memory is neglectable.

### Realization of a fast search on human chromosome

Our original motivation for developing RScan is to realize a fast similarity search for the structured RNAs in a large genome using a PC. The difficulties arise from the limitations of the storage and speed of PC. Here, four pre-miRNAs and human chromosome 21 were used as an example to show how to apply RScan to search a large genome database. The hash table of the structural database of chr21 genome sequence that was divided by a 150 nt slide window was generated in advance and *k *was set to be 9 or 11 while the match-core was 3 times of *k*. RScan took less than 4 minutes on a PC for searching each pre-miRNA in chr21, shown in Table 6. In all cases, RScan quickly found the query miRNA-self and its structural similarities, which can be further evaluate if they are miRNA candidates based on the characteristics of the miRNA. But these structural similarities did not contain other known miRNAs. If we set more loose parameters, *k *was 7 and the match core was 2 times of *k*, the query "hsa-mir-155" would align all other three miRNAs. Of course, this would spend more time and find thousands of structural similarities.

**Table 6:** Search the pre-miRNAs in human chromosome 21

**Query**	**Database**	**k = 9**		**k = 11**	
			
		**Hits/Known^1^**	**T_search _(s)**	**Hits/Known**	**T_search _(s)**
hsa-mir-99a	150 nt chr21 structural database	24/1	234	3/1	71
hsa-let-7c		6/1	151	2/1	49
hsa-mir-125b-2		12/1	139	3/1	38
hsa-mir-155		14/1	228	4/1	66

^**1 **^Known: number of the known miRNAs matched by hits.

In addition, we used the member of the let-7 family, "has-let-7a-2", which is located in human chromosome 11 and has 88% sequence similarity with the "has-let-7c" located in chr21, as a query to search its structural similarities in chr21. Parameters *k *was set to 9 and the match core was 2 times of *k*, RScan found seven similarities, including his homology miRNA "has-let-7c", and spent about a thousand seconds.

In real-life applications, an additional step could be considered for reducing the storage of large genome. We may use RNALfold [13] to find the stable structures or significant local structural motifs in the genome, which are then used to build the structural database. RScan could get more significant structural alignments on this filtered structural database. To sum up, this experiment shows that RScan is competent in the real-life applications of searching structural similarities for structural RNAs in large genome.

### Real-life application for validating the unknown RNA sequence

Given an unknown query, to determine if it belongs to known ncRNA families, we can run a structural similarity search on entire ncRNA database, like Rfam, by RScan. Suppose that the AB042240.3/15036-15107, which is the tRNA query mentioned in previous experiments, is the unknown query. RScan can use the parameters with very strict thresholds to quickly search similar structures of the query in Rfam seed structural database (see Methods). RScan only ran 38 seconds to report 216 significant alignments when the match-core was 21 (Table 7). And 206 out of 216 hits were the members of tRNA family. So, the query will be validated as the tRNA family. It can be clearly seen that RScan provides the user with a quick understanding of the query. Moreover, RScan can perform subtler searches using relaxed the match-core, like 14. However this would take more time and would get more tRNA hits. Table 7 lists RScan's search results for the six ncRNA queries mentioned in previous experiments. The user can choose strict or loose RScan parameters for balancing the computation time and more alignment results.

**Table 7:** Search RNA sequences in Rfam seed structural database

**Rfam ID of Query**	**RScan Parameters^1^**				**Hits/ST/F5^2^**	**T_search _(s)**
			
	**k**	**Mc**	**Id ≥ %**	**Ar ≥ %**		
RF00005/AB042240.3/15036-15107	7	21	90	95	216/206/5	38
RF00001/M28193.1/1-119	7	21	90	95	72/70/5	79
RF00030/AY090594.1/1-274	9	27	80	95	2/2/2	140
RF00011/U64878.1/1-308	9	27	80	95	3/3/3	65
RF00010/U28101.1/1-327	9	27	80	95	18/16/5	42
RF00177/AF050603.1/1-475	9	27	80	95	74/74/5	884

^**1 **^Mc: match-core; Id: identity; Ar: Alignment ratio.

^**2 **^ST: number of same type of ncRNA with the query; F5: number of same type ncRNA in first 5 hits.

We then employed a larger sample of query sequences. Table 8 shows that ten ncRNA queries, selected randomly from the entire Rfam seed database, and the above six RNA families were just excluding. RScan searched structural similarities for them in the Rfam seed database and most queries were recognized. Unfortunately the RF00009/AF004373.1/1-320 and RF00436/AL591676.10/16205-16259 were missed. For RF00009/AF004373.1/1-320, this might be relative to the low stability of the query's predicted secondary structure. For RF00436/AL591676.10/16205-16259, it was too short and formed only one short stem-loop. This simple and general secondary structure aligned numerous false positive hits. Generally, according to "Mp" and "QL" columns in Table 8, RScan had a good performance on the query with unstable structure or the short query such as RF00181/AL132709.5/131508-131439 or RF00480/AY455785.1/1517-1568. Consequently, RScan is competent for the task of searching structural RNA in Rfam.

**Table 8:** Search more RNA sequences in Rfam seed structural database

**Rfam ID of Query**	**Parameters^1^**				**Hits/ST/F5^2^**	**T_search _(s)**	**Mp^3^**	**QL^4^**
					
	**k**	**Mc**	**Id**	**Ar**				
RF00009/AF004373.1/1-320	9	27	80	95	1/1/1	10	0.426	320
RF00048/AF405669.1/4445-4505	7	21	90	95	4/3/3	3	0.013	61
RF00167/AL591981.1/205922-205823	7	21	90	95	3/3/3	41	0.140	100
RF00175/AF042101.1/695-812	7	21	90	95	64/64/5	44	0.205	118
RF00181/AL132709.5/131508-131439	7	21	90	95	5/5/5	6	0.886	70
RF00229/AY184219.1/389-639	9	27	80	95	21/21/5	35	0.314	251
RF00342/AP001273.4/3902-3830	7	21	90	95	6/5/5	4	0.013	73
RF00376/AY451114.1/148-231	7	21	90	95	248/248/5	22	0.013	84
RF00436/AL591676.10/16205-16259	7	21	90	95	31/2/2	27	0.062	55
RF00480/AY455785.1/1517-1568	7	21	90	95	453/427/5	10	0.025	52

^**1 **^Mc: match-core; Id: identity ≥ %; Ar: Alignment ratio ≥ %.

^**2 **^ST: number of same type of ncRNA with the query; F5: number of same type ncRNA in first 5 hits.

^**3 **^Mp: p-value of minimum free energy of queries' predicted secondary structures.

^**4 **^QL: length of query.

In this real-life application, RScan is very convenient for the researchers who might produce or obtain numerous transcripts by biological experiments and would want to know if some of them have similar structures with known ncRNA and also to deduce their possible functions. According to Table 7 and 8, if an unknown sequence is perfectly aligned an ncRNA, it can be assigned as the same type. Now, we are building RScan as a web server for supporting more ncRNA structural databases from experimental and computational databases, like RNAdb, NONCODE, Fantom3, etc.

## Discussion

RScan is a fast and sensitive algorithm for searching RNA secondary structure similarity and it is valuable for real-life applications. RScan begins the process by converting the sequence database into a structural database. RScan can then search a structural query on the structural database to obtain the optimal structural alignments. To increase search efficiency, RScan employs a hash table to store *k*-tuples of the structural database. Consequent searches identify all possible match-cores based on the hash table, and score candidate alignments derived from the match-cores to obtain optimal alignments. It should be emphasized that the structural database and the corresponding hash tables are all generated only once and this drastically reduces the search time.

When a database is for a single genome, RScan slides along the genome sequence with a window of predetermined length to segment sequences. The structural database is then created from the predicted secondary structures of these segmented sequences. In traditional methods, a query is only aligned with the sub-sequences of the genome; these sub-sequences are generally less than the maximum length [13,18]. So, using RScan to transform the genome into a structural database is sensible. However, a PC cannot afford the huge storage required for building a structural database with all lengths. In our example, we used a 150 nt window, which slides along the both strands of chromosome 21 stepped every 50 nt, to build the structural database. Notice that this sliding window multiplies the genome data six times and it is helpful to prevent the searching from loss of sensitivity. Considering the substantial memory usage of the hash table using structural database to reduce memory requirement becomes markedly relevant. In further application, an additional step may be considered for reducing the storage and improving search performance. We may use RNALfold [13] to find the stable structures or significant local structural motifs in the genome, which are then used to build the structural database. RScan could perform more efficiently on this filtered structural database and the structural alignments could be more significant.

As highlighted by Klein and Eddy, three areas demand additional analysis: the score matrix, the precise secondary structure of the query sequence, and the speed [1]. Since the score matrix is independent of the alignment algorithm, RScan is not involved in refining the score matrix. RScan only uses the simplest match\unmatch score function. In terms of the query sequence, RScan utilizes known or predicted secondary structures, much like the strategy used by RSEARCH. In most cases, the correct secondary structures of a sequence are difficult to obtain [1]. In order to acquire a good secondary structure, all possible folds of the sequence are considered in Sankoff's algorithm [27]. However, the optimal energy structure may not necessarily be the correct structure. In such a case considering all possible folds will substantially be slower than many other methods. Some of the methods use various constraints to reduce the required folds to predict secondary structures [1]. RScan employs RNAfold to predict the secondary structures of queries and segmented sequences. This means that only a certain secondary structure of a sequence is considered. This implies that the efficiency of RScan greatly depends on the accuracy of the secondary structure prediction algorithm. Improving the score matrix and secondary structure prediction will be considered in our future work, and updates in these areas can be modularly integrated into RScan. In addition, RScan does not evaluate the statistical significance, the P-value, for each alignment. The P-value is generally calculated based on the size of the database and the composition of the sequences in the database [1]. Since secondary structures in the structural database are generated via prediction, the composition of the structural symbols is not sufficiently credible for a statistical evaluation. Finally, RScan focuses on improving search speed, especially on search of large-scale databases. Essentially, RScan transfers the complicated similarity search problem of RNA secondary structure into a sequence alignment issue between a structural query and a structural database. So, the time complexity of RScan is *O*(*NM*), identical to that of the sequence alignment algorithms. RScan successfully realizes a quick search of similar secondary structures for the structured RNAs in large databases. In the future, our aim is to build an online RScan server for the applications used by biology and bioinformatics researchers.

## Conclusion

RScan can find structural similarities for structured query RNAs in large databases efficiently and quickly. It is a preferable choice for real-life application of structural alignment.

## Methods

### Non-coding RNA family datasets from Rfam

Six ncRNA families: the tRNA family (Rfam number is RF00005), two rRNA families (RF00001 and RF00177) and three RNase P families (RF00010, RF00011 and RF00030), were extracted from Rfam database, version 7.0 [25]. The members of each family were selected carefully for filtering the sequences with sequence similarity or with unstable predicted structures or with various secondary structures. We used the tRNA family as an example to explain the filtering criteria. Firstly, BLASTCLUST [24] was used with parameters: S = 80, L = 0.5, W = 16 to calculate the sequence similarity of the tRNA members. Only one member in each cluster was kept. Secondly, the method reported by Bonnet *et al*. [28] was used to analyze the stability of the secondary structure of each kept member sequence. P-values of the free energies of the secondary structures were then calculated based on each sequence and their 1000 shuffling sequences with invariable di-nucleotide frequency. If the p-value was lower than 0.05, the corresponding sequences were kept. This step can obtain the member sequences with quite stable secondary structures and decrease inaccuracy from secondary structure prediction software. Thirdly, the shapes of the predicted secondary structures of member sequences were also limited. According to the common understanding, the tRNA can form a typical cloverleaf structure. However, RNAfold predicted diverse secondary structures for tRNA members. So, only the members' predicted secondary structures with 3 loops were collected. Use of the above criteria, we finally collected 67 tRNAs as the tRNA dataset. For other five ncRNA families, the same criteria were also used for building corresponding datasets. For different ncRNA family, we limited the number of loops that most frequently occurred in the corresponding family's predicted structures. In addition, the lengths of sequences in rRNA RF00177 family were from 195 nucleotides to 832 nucleotides. So, we further limited the length range of this family from 450 nt to 550 nt. The basic information of six ncRNA datasets was listed in Table 9.

**Table 9:** The basic information of filtered ncRNA family datasets

**Family dataset**	**Description**	**# of members**	**Average Length**	**Limited Loops**
tRNA/RF00005	tRNA	67	74	3
rRNA/RF00001	5S ribosomal RNA	25	118	2
rRNA/RF00177	Small subunit ribosomal RNA	35	518	11,12
ribozyme/RF00010	Bacterial RNase P class A	53	358	7,8,9
ribozyme/RF00011	Bacterial RNase P class B	8	372	8,9
ribozyme/RF00030	RNase MRP	9	291	6,7

For each filtered dataset, one member was used as the query and other members were used as the database. For RScan search, the secondary structures of the query and the sequences in the database were predicted by using RNAfold [23] as the structural query and the structural database, respectively. The parameters of RScan were set at: *k *= 7, Identity ≥ 90% and Alignment_ratio ≥ 95% for tRNA (whose lengths were less than 100 nt), and *k *= 7, Identity ≥ 85% and Alignment_ratio ≥ 90% for RF00001 (whose length range were from 100 nt to 200 nt), and *k *= 9, Identity ≥ 75% and Alignment_ratio ≥ 85% for other families (whose lengths were more than 200 nt). For RSEARCH search, all sequences in the database were jointed as a single sequence and RSEARCH searched the query in the jointed sequence with the parameters "-n 1000 -E 10". All experiments were carried out on a 2.4 GHz Intel PC with 1 GB of RAM, running Linux.

### Randomized genome

The *A. pernix *genome (NC_000854.1, 1.67 Mb) was taken from NCBI. It was shuffled with an identical di-nucleotide frequency to create a shuffling genome. Then, 49 tRNAs and 20 rRNAs recognized by both RSEARCH and RScan according to the ncRNA dataset experiments were inserted in the shuffling genome to make two randomized genomes. The queries tRNA "AB042240.3/15036-15107" and rRNA "M28193.1/1-119" in Table 1 were used again.

For RScan search, the randomized genome inserted with tRNAs was broken into 75-nucleotide (75 nt) segment sequences because the query tRNA was 72 nucleotides. Every segment sequence was forced to contain unknown character "N" less then 5%. The secondary structures of all 75 nt segment sequences were predicted using RNAfold [23]. The set of all symbol sequences of secondary structures comprised the structural database. The randomized genome inserted with rRNAs was processed with the same steps except that it was broken into 120-nucleotide segment sequences because the query rRNA is 119 nucleotides.

### Rfam seed structural database

This database came from Rfam version 7.0, seed alignments of 503 families, which contained 13040 seed sequences [25,29]. The secondary structures of all sequences were predicted using RNAfold [23]. For RScan search, the hash tables of the structural database were generated in advance. The *k*-tuple was set to 7 or 9 and the storages of the hash table were 2.02 Mb or 1.58 Mb.

### Structural database of human 21 chromosome

The human chromosome 21 (chr21) was downloaded from NCBI, totally 45.6 Mb. A 150 nt slide window stepped every 50 nt along both strands of the human chr21 to produce 1,366,746 segment sequences. The secondary structures of all sequences were predicted using RNAfold [23] to build structural database. The hash tables of the structural database were also generated in advance and the *k*-tuple was set to 9 or 11. The storages of the hash table were 172 Mb or 145 Mb, respectively. Moreover, the hash table was technically divided into small files, which were read in memory one by one. This maintained a limited requirement for computer memory (see supplementary materials). The query sequences were four pre-miRNAs locating in human chromosome 21, taken from the microRNA registry [30].

## Availability and requirements

The RScan program is freely accessible on our website . Supplementary materials, the detailed data of the experiments and the recommended parameter settings are also provided at the website.

## Competing interests

The author(s) declare that they have no competing interests.

## Authors' contributions

CX initiated the project and developed the method and implemented all of the experiments. CX wrote the manuscript. GPL provided helpful insights and helped with the writing of this paper.
